# Open Notes in Swedish Psychiatric Care (Part 1): Survey Among Psychiatric Care Professionals

**DOI:** 10.2196/mental.9140

**Published:** 2018-02-02

**Authors:** Lena Petersson, Gudbjörg Erlingsdóttir

**Affiliations:** ^1^ Faculty of Engineering Department of Design Sciences Lund University Lund Sweden

**Keywords:** electronic health record, eHealth, baseline survey, mental health, open notes, psychiatry, health professionals

## Abstract

**Background:**

When the Swedish version of Open Notes, an electronic health record (EHR) service that allows patients online access, was introduced in hospitals, primary care, and specialized care in 2012, psychiatric care was exempt. This was because psychiatric notes were considered too sensitive for patient access. However, as the first region in Sweden, Region Skåne added adult psychiatry to its Open Notes service in 2015. This made it possible to carry out a unique baseline study to investigate how different health care professionals (HCPs) in adult psychiatric care in the region expect Open Notes to impact their patients and their practice. This is the first of two papers about the implementation of Open Notes in adult psychiatric care in Region Skåne.

**Objective:**

The objective of this study was to describe, compare, and discuss how different HCPs in adult psychiatric care in Region Skåne expect Open Notes to impact their patients and their own practice.

**Methods:**

A full population Web-based questionnaire was distributed to psychiatric care professionals in Region Skåne in late 2015. The response rate was 28.86% (871/3017). Analyses show that the respondents were representative of the staff as a whole. A statistical analysis examined the relationships between different professionals and attitudes to the Open Notes service.

**Results:**

The results show that the psychiatric HCPs are generally of the opinion that the service would affect their own practice and their patients negatively. The most striking result was that more than 60% of both doctors (80/132, 60.6%) and psychologists (55/90, 61%) were concerned that they would be less candid in their documentation in the future.

**Conclusions:**

Open Notes can increase the transparency between patients and psychiatric HCPs because patients are able to access their EHRs online without delay and thus, can read notes that have not yet been approved by the responsible HCP. This may be one explanation as to why HCPs are concerned that the service will affect both their own work and their patients.

## Introduction

### The Development of Open Notes

A discussion has surfaced recently about the effects of patients having online access to their electronic health records (EHRs; here referred to as Open Notes) in psychiatric care [1,2] and whether patients and health care professionals (HCPs) would be put at risk by the service [3]. However, not many psychiatric care clinics have implemented such a service. According to Open Notes Mental Health (toolkit), there are 21 implementations to date in the United States and Canada, apart from the seven regions that have implemented the service in psychiatry in Sweden. As all these implementations are recent, little is known about the perceptions of HCPs to the service in this context.

Open Notes is one of the most important civic electronic health (eHealth) services in Sweden. All citizens in the country are able to access their EHRs from care online and can thus read clinical notes through the Internet. The initial implementation of the service in hospitals, primary care, and specialized care had previously raised both questions and resistance among the HCPs [4-6]. However, no baseline studies were conducted at that time that can be compared with the one presented in this paper. When the service was launched in 2012 in Sweden, some medical specialties, where patient digital access was considered sensitive, were exempt. One of these was psychiatry. In 2015, Region Skåne, the county board of the southernmost county in Sweden was the first in the country to add adult psychiatry to Open Notes. Through the service, patients in adult psychiatric care in Region Skåne were able to access entries in their EHRs from September 5, 2015 online. This development is in line with the reasoning of the OpenNotes Project in the United States—that patients in psychiatric care should not be treated differently than other groups of patients [1,3,7]. The introduction of Open Notes in psychiatry provided an opportunity for us to carry out a unique baseline study by conducting a full population survey of the employees in adult psychiatry in Region Skåne before the service became available to patients.

### The Technical Prerequisite

In Region Skåne, in contrast to the OpenNotes Project and the system used by the US Department of Veterans Affairs (VA), patients can access their EHRs as soon as they are entered into the system and can thus, read their clinical notes in many cases before the responsible HCP has signed off on them. This means that the notes have not yet been approved by the responsible HCP when they are made available for the patient to read online. On the other hand, a signed note means that the responsible HCP has decided that the information is correct. HCPs can neither opt out from participating nor can they choose which patients can access the service. This is because the service has been implemented in the entire public health care system in the region and thus, includes all inpatients and outpatients.

The Open Notes service in Sweden is accessible by logging into a secure online patient portal. In certain cases, information is withheld from patients, such as information that could pose risks to the patient or relatives. To ensure the ability to enter such information in the health record, there is a special template for this purpose called specific information. This information is only digitally accessible to the HCPs, but patients can access it by requesting a paper copy of their EHRs. In Region Skåne, inpatients in adult psychiatric care (approximately 5% of the patients) are exempted from immediate access to the service but can access their EHRs 4 weeks after hospitalization. The rationale for this decision is the risk that inpatients will read their Open Notes at a critical stage in their treatment and that this could harm them. There is also the risk that inpatients would compare their notes with those of other inpatients, become upset, and agitate each other when they find differences in the treatment. Outpatients in psychiatric care can read their entries right away, just as patients in non-Psychiatric care in Region Skåne have been able to do since the service was first introduced.

The aim of this study was to describe, compare, and discuss how different HCPs in adult psychiatric care in Region Skåne expect Open Notes to impact their patients and their own practice. This is the first of two papers about the implementation of Open Notes in adult psychiatric care in Region Skåne. The second one (in preparation) will report on the actual experiences of the HCPs.

## Methods

### Survey Design

The material presented is the product of a baseline survey in psychiatric care. This is a substudy in a research project (the EPSA Project, financed by AFA Insurance in Sweden) on how the work and work environment of HCPs are affected by civic eHealth services such as Open Notes.

The baseline survey used in this study is based on one developed and implemented by the OpenNotes Project in the United States [8-10]. In line with the original survey [8,11], the Swedish version covers three areas: *The impact on the patients*, *The impact on the practice*, and *About me*. First, the original OpenNotes survey was translated and adjusted to fit the Swedish context. Second, the researchers conducted four multiprofessional focus groups with employees. The purpose was to validate the areas of interest in the questionnaire in the Swedish context. Third, a Web survey was designed concerning online patient access to their EHRs and the work environment of the HCPs who meet the patients. Previous surveys on the implementation of online patient access to their EHRs in Sweden have been directed to either doctors or nurses [5]. The survey consisted of 34 fixed-choice questions (mostly 4-point scale answers) and three open-ended questions and was designed so that the respondents could choose not to answer all the questions.

### Setting and Population

The Division of Psychiatric Care in Region Skåne consists of three subdivisions: adult, children and youth, and forensic. It was decided that only patients in adult psychiatry would be offered online access to their EHRs to begin with. The adult psychiatry subdivision employs roughly 3000 people. In 2013, there were 436,000 outpatient visits and 6600 inpatients in adult psychiatric care in the region. Online access to the EHR service for all adult psychiatric care patients opened on October 5, 2015.

The entire population of HCPs (n=3017) in adult psychiatry in Region Skåne who meet patients were invited to participate in this study. This included assistant nurses, doctors, medical secretaries, nurses, occupational therapists, physical therapists, psychologists, and social workers. The rationale for not taking a sample was that it is a heterogeneous population where some of the professional groups are large and others are small. In addition, the employees in Region Skåne were the first in psychiatric care in Sweden whose patients would be able to read their notes online, and thus, it was important that everyone in the population had the opportunity to answer the survey.

### Survey Administration

We used the Web survey tool, *Sunet Survey*. The emails were sent from Lund University. On September 17, 2015, a prenotification email was sent to the study population, and on September 18, the survey was sent electronically to the institutional email addresses of the professionals with a cover letter and a link to the survey. Both the prenotification email and cover letter informed the recipients that participation was voluntary, that the computer files with the results were confidential, respondents could terminate their participation at any time, and tracking of individual responses was not possible. We did not offer any survey incentives. We sent four reminders, and the survey closed on October 2, 3 days before patients were given online access to their EHRs. All the material in the baseline study was thus collected before the implementation.

### Data Analysis

We present descriptive information for each fixed-choice question and chi-square tests to examine the relationships between professionals and attitudes to the Open Notes service. Due to the small number of respondents, occupational therapists, physical therapists, social workers, and all others (as one group) were grouped together for the chi-square tests. All reported *P* values were two-sided. *P*<.05 was considered statistically significant. Due to the answer options, we did not conduct chi-square tests on five of the questions. The survey data was imported and analyzed in Statistical Package for the Social Sciences (SPSS) version 23 (IBM Corp). The analysis of the open-ended questions is not included in this paper.

### Ethics

The researchers followed the guidelines on research ethics issued by the Swedish Research Council [12]. This study does not cover any sensitive information and does not require ethical approval according to the Swedish regulations on research ethics.

## Results

### The Respondents

The response rate to the Web survey was 28.86% (871/3017). The distribution between the different professions corresponds well with the overall percentage of employees in each profession in the region. The questionnaire was distributed to both permanent and temporary employees, which may have influenced the response rate negatively. Table 1 presents the demographics of the survey respondents. For statements that evaluated attitudes, we combined the alternatives somewhat agree and agree, indicating that the respondent agreed to some level. Tables 2 and 3 provide more information about this process.

### Expected Impact on Patients

Table 2 presents the percentages of respondents who somewhat agree or agree with questions about the impact of the service on the patients. They are generally pessimistic about the future impact of Open Notes on patients. Almost 58.0% (488/840) believe that their patients will worry more after reading their notes, and only 11.2% (93/833) believe that the service will inspire their patients to take better care of themselves. More than 63.2% (529/837) expect that their patients will disagree with the content in their notes, and half of the respondents (436/832) expect their patients to request changes in the notes. Only 27.4% (227/830) of the respondents believe that Open Notes will increase the patients’ trust for them as professionals.

The chi-square tests show that there are differences in opinions among the different groups of professionals, especially regarding whether patients will be satisfied with the content in their notes and if Open Notes will increase the patients’ trust for the HCPs. Medical secretaries (55/72, 76%) and assistant nurses (126/172, 73.3%) agree to a larger degree with the statement, A majority of patients will disagree with what is written in their notes, than doctors (82/131, 62.6%) and psychologists (44/89, 49%). Medical secretaries (46/68, 68%) agree to a larger degree with the statement, A majority of patients will request changes to the content of notes, compared with doctors (77/128, 60.2%) and psychologists (35/89, 39%). Doctors (24/130, 18.5%) and psychologists (16/89, 18%), in turn, agree with the statement, A majority of patients will trust me more as their caregiver, to a lesser degree than nurses (59/217, 27.2%) and assistant nurses (58/178, 32.6%).

**Table 1:** Demographic characteristics of the respondents in percentage and number (n). A total of 853 of the 871 respondents answered the professional affiliation question; 851 answered the gender question.

Professional affiliation and gender		Survey respondents, n (%)
Nurse		228 (26.7)
Assistant nurse		182 (21.3)
Doctor		133 (15.6)
Psychologist		91 (10.7)
Medical secretary		76 (8.9)
Social Worker		57 (6.7)
Other		53 (6.2)
Occupational therapist		17 (1.9)
Physical therapist		16 (1.9)
**Gender**		
	Female	628 (73.8)
	Male	223 (26.2)

**Table 2:** Psychiatric professionals’ views on how patient online access to EHRs in adult psychiatric care will affect the patients: proportion of respondents who *somewhat agree* to *agree* and the results of the chi-square test for these items. The number of total responses for each item ranged from 830 to 840.

Survey item		n (%)	*P* value
**Among my patients who read their electronic health record from psychiatry online**			
	A majority of patients will better understand their health and medical conditions.	252 (30.1)	.36
	A majority of patients will worry more.	488 (58.1)	.32
	A majority of patients will better remember the plan for their care.	402 (48.2)	.63
	A majority of patients will disagree with what is written in their notes.	529 (63.2)	<.001
	A majority of patients will request changes to the content of notes.	436 (52.4)	.001
	A majority of patients will take better care of themselves.	93 (11.2)	.15
	A majority of patients will be more likely to take medications as prescribed.	154 (18.5)	.13
	A majority of patients will find significant errors in the notes.	351 (41.9)	.03
	A majority of patients will feel more in control of their health care.	372 (44.4)	.53
	A majority of patients will be better prepared for visits.	261 (31.1)	.02
	A majority of patients will trust me more as their caregiver.	227 (27.4)	.001
	A majority of patients will contact me or my practice with questions about their notes.	570 (68.7)	.002
	A majority of patients will find the notes to be more confusing than helpful.	438 (52.7)	.03

^a^Represents the number and percent of respondents who indicated *somewhat agree* to *agree* on a 4-point scale with the following response options: *disagree*, *somewhat disagree*, *somewhat agree*, and *agree*.

### Expected Impact on Practice

Table 3 shows how the respondents expect Open Notes to affect their practice, way of documenting, and care delivery. Approximately 40% of the respondents believe that visits will take longer (299/852), that they will have to take care of patients’ questions in addition to the visits (343/845), and that patients will be offended when they read their notes online (376/844). Forty percent (342/845) expect to become less candid in their documentation and that they will spend significantly more time writing, dictating, or editing notes. Only 21.2% (177/838) believe that care delivery will become more efficient. Approximately one-third expect (305/839) an increase in patient safety. Seventeen percent (147/850) think that the service will contribute to care on equal terms for all patients to a large or very large extent. Thirty-six percent (302/849) believe that the relationship between their profession and the patient will change, but only 20.6% (174/845) think that relationships between different professions in adult psychiatric will change. Nearly half of the respondents (386/846) believe that the implementation of Open Notes increases the risk for threat and violence. Very few of the respondents (75/851, 8.8%) often meet patients who have read their health record on paper. Approximately one-third (231/835) of the respondents agreed that Open Notes in adult psychiatric care is generally a good idea.

The chi-square test results in Table 3 show that there are differences among the professional groups, especially on the items that are about clinical documentation and the general idea of Open Notes. Doctors (63/133, 47.4%) and medical secretaries (31/71, 44%) are more worried that visits will take more time compared with nurses (71/228, 31.1%) and psychologists (21/90, 23%). The results show the same pattern when it comes to the statement I will spend significantly more time addressing patient questions outside of visits. Approximately half of the doctors (73/132, 55.3%) and medical secretaries (34/69, 49%) claim that they are moderately concerned, very concerned, or so concerned that they do not want Open Notes to be implemented in psychiatric care at all compared with nurses (86/227, 37.9%) and psychologists (32/89, 36%).

Medical secretaries (42/69, 61%) and doctors (73/132, 55.3%) are also more worried about patients being offended when reading their notes online than are assistant nurses (82/178, 46.1%), nurses (94/227, 41.4%), and psychologists (36/90, 40%). Sixty one percent of both doctors (80/132) and psychologists (55/90) are worried that they will be less candid in their documentation. More than half of the doctors (76/133, 57.1%) and psychologists (46/90, 51%) and 45% (31/69) of the medical secretaries are worried that they will have to spend significantly more time writing, dictating, or editing notes. Finally, doctors (25/132, 18.9%) and psychologists (17/87, 20%) are less likely to agree with the statement Patient online access to their EHRs in adult psychiatry is generally a good idea than medical secretaries (19/72, 26%), nurses (60/223, 26.9%), and assistant nurses (51/175, 29.1%).

In summary, the results show that the HCPs were of the opinion that the service will negatively impact their work in different ways. The statistical analysis also shows that people in different professional groups vary concerning their misgivings about how the service will affect their own work: doctors, psychologists, and medical secretaries in many cases are more negative to the service than the other professional groups.

**Table 3:** Psychiatric professionals’ views on patients’ online access to electronic health records (EHRs) in adult psychiatric care: expectations of how practice and clinical documentation will be affected and the results of the chi-square test for these items. The number of total responses for each item ranged from 835 to 852.

Survey item		n (%)	*P* value
Visits will take significantly longer^a^		299 (35.09)	<.001
I will spend significantly more time addressing patient questions outside of visits^a^		343 (40.6)	<.001
Patients who read their notes will be offended^a^		376 (44.5)	<.001
I will be less candid in my documentation^a^		342 (40.5)	<.001
I will spend significantly more time writing or dictating or editing notes^a^		352 (41.5)	<.001
Medical care will be delivered more efficiently (yes response)^b^		177 (21.1)	.008
Patient satisfaction will improve (yes response)^b^		247 (29.5)	.06
Patient care will be safer (yes response)^b^		305 (36.3)	.01
Patient online access to their EHRs^c^will contribute to health care on equal terms for all patients (large or very large extent)^d^		147 (17.3)	.01
Patient online access to their EHRs in adult psychiatry will affect the relationship between the different professionals working there (large or very large extent)^d^		174 (20.6)	.006
Patient online access to their EHRs in adult psychiatry will affect the relationship between the patient and your profession (large or very large extent)^d^		302 (35.6)	.02
Patient online access to their EHRs in adult psychiatry will affect the risk for me to be subjected to threat and violence (will increase)^e^		386 (45.6)	
Patient online access to their EHRs in adult psychiatry will affect the risk for me to be reported to the Patients Advisory Committee (will increase)^e^		356 (42.2)	
Patient online access to their EHRs in adult psychiatry will affect the risk for me to be reported to Health and Social Care Inspection (will increase)^e^		273 (32.2)	
How often do you meet patients who have read their health record on paper? (to a large or a very large extent)?^f^		75 (8.8)	
**How many of your patients do you think will read their EHRs online?**^g^			
	0-10 (%)	121 (14.4)	
	11-25 (%)	214 (25.4)	
	26-50 (%)	250 (29.7)	
	51-75 (%)	196 (23.3)	
	76-100 (%)	60 (7.1)	
Patient online access to their EHRs in adult psychiatry is generally a good idea (somewhat agree to agree)^h^		231 (27.7)	<.001

^a^Number and percentage of employees responding that they were *moderately concerned*, *very concerned*, or *so concerned that I do not want patient online access to my EHR in psychiatric care at all*. It was also possible to choose the options *minimally concerned* and *not concerned*.

^b^Number and percentage of employees responding *yes*. It was also possible to answer *no*.

^c^EHR: electronic health record.

^d^Number and percentage of employees responding that they to *a large extent* or *a very large extent* agree. It was also possible to choose the options to *a little extent* or *not at all*.

^e^Number and percentage of employees responding that *the risk will increase*. It was also possible to answer *the risk will not change*, *the risk will decrease*, and *not relevant*. Due to the answer options, we did not conduct a chi-square test on these questions.

^f^Number and percentage of employees responding that they to *a large extent* or *a very large extent* agree. It was also possible to choose the options to *a little extent*, *not at all*, or *not relevant*. Due to the answer options, we did not conduct a chi-square test on this question.

^g^Due to the answer options, we did not conduct a chi-square test on this question.

^h^Represents the number and percent of respondents who indicated *somewhat agree* to *agree* on a 4-point scale with the following response options: *disagree*, *somewhat disagree*, *somewhat agree*, and *agree*.

## Discussion

### Principal Findings

To our knowledge, this is the first baseline study that examines how HCPs working in adult psychiatric care in public care expect Open Notes to affect their work and how these expectations vary between different professions. Generally though, the respondents in this study are more negative to Open Notes than the respondents in previous baseline studies in non-Psychiatric settings in the United States [3,8]. As the service is obligatory in Sweden, HCPs cannot opt out from participating, and it is not possible to exclude patients. In the US OpenNotes Project, doctors were enrolled on a voluntary basis and could exclude patients they thought were less apt to handle the service. This might account for some of the differences. Another explanation may be that transparency in psychiatric care can be more problematic as the content in the notes may worry patients and could influence the patient-doctor relationship [13].

An important difference, compared with similar services in the United States [11,14], is that Region Skåne clinical notes are accessible to outpatients without delay. This has been one of the most debated features of the Open Notes service in Sweden, even in non-Psychiatric settings, because it allows patients to read entries in their EHRs before they are signed [4]. This may explain why approximately 60% of both doctors and psychologists are worried that they will be less candid in their documentation in the future. The OpenNotes Project in the United States has also expressed concerns that increased transparency may *water down* the content of the records [2]. Furthermore, in a recent study of Open Notes at the VA System in the United States, mental health clinicians claim that they are more careful about what they write to protect the patients and themselves [15].

Many of the respondents are pessimistic in their expectations of the impact of Open Notes on their patients in adult psychiatry. There is a need for more knowledge about the effects of the service on patients in general [16]; in psychiatric care, it may be particularly important to gain a greater understanding of how the service affects patient groups with different diagnoses.

This is the first study where medical secretaries are asked about their expectations regarding the implementation of Open Notes. Medical secretaries work with administrative tasks such as transcribing dictated notes, talking to patients on the telephone, and meeting them at the reception. To our surprise, the chi-square tests show that in some cases, medical secretaries are as negative as the doctors. Seventy-six percent believe that a majority of the patients will disagree with the content of their records, and over 60% answered that they were worried that patients who read their notes online will be offended by the entries. The medical secretaries may be concerned that because they are working on the front line, they will be the ones who will first encounter the disappointed and perhaps upset patients.

Region Skåne has been informed about the results from the baseline survey and is aware of the possible implications of the deployment of the Open Notes service. It will consider taking actions when the results from the follow-up survey are fully analyzed in the spring of 2018. The results of this baseline study are being used to influence the planned implementation of Open Notes in children and youth psychiatry and forensic psychiatry in Region Skåne.

### Limitations

This study has a number of limitations. First, the response rate to the Web questionnaire was 28.86%. One explanation may be that this is a full population study, and some employees did not work during the time when it was possible to answer the survey. Still, the group distribution among the respondents corresponds well with the percentage of employees in each profession, which indicates that we have good representation of all professional groups. Second, the topic of a Web survey can affect the response rate among respondents [17]. On the one hand, a topic of high interest to respondents can increase the response rate; on the other, topics of a sensitive nature may result in a lower response rate [17]. The rhetoric put forth by key actors in Sweden is that Open Notes is a civic service and a technical solution aimed at the patient. Thus, any significant impact that it may have on the work or work environment of HCPs has not been taken into consideration. However, we know from our previous studies in non-Psychiatric settings that Open Notes has raised both questions and resistance among HCPs in Sweden [4]. Thus, the topic of the questionnaire is sensitive, and this may have affected the response rate negatively. Third, the results are limited to only one out of 20 regions in Sweden. Region Skåne, however, is one of the three largest regions in Sweden and was the first to implement the Open Notes service in psychiatric care, which made the study possible.

### Conclusions

Over 40 years ago, Shenkin and Warner presented the iconoclastic idea of patient access to their records on a regular basis [18]. The vision of the authors was that this would enhance the quality of care. For less than a decade now, digitalization in health care has made it technically possible to provide patients with online access to their records, and the reactions to the service may not be fully as positive as Shenkin and Warner anticipated. The results of this study show that the HCPs in psychiatric care in Region Skåne expect the implementation of patient access to their EHRs to have mainly a negative impact on their patients and on their own working life. The main concern seems to be linked to the enhanced transparency that the service offers to the patients. The fact that roughly 60% of the doctors and psychologists state that they will change their entries as a result of the implementation is alarming. Not only does this indicate that the Open Notes service will affect the working life of the doctors and psychologists but also that the service may not meet the intentions of the implementers, that is, to provide patients with full information about their health conditions. Many questions about the factual impact of the service in psychiatric care still remain unanswered. During the spring of 2017, we distributed a follow-up survey to the employees in adult psychiatric in Region Skåne. We hope to be able to answer some of these questions in the next paper by comparing the results from the baseline study presented here with the results from the follow-up survey. Thus, the second paper will report on the actual experiences of the HCPs.

## Abbreviations

eHealth: electronic health
EHR: electronic health record
HCP: health care professional
VA: Veterans Affairs
